# Derivation and Differentiation of Adipose-Tissue Regulatory T Cells: A Stepwise, Multi-Site Process

**DOI:** 10.3389/fimmu.2020.599277

**Published:** 2020-10-29

**Authors:** Pulavendran Sivasami, Chaoran Li

**Affiliations:** Department of Microbiology and Immunology, Emory University School of Medicine, Atlanta, GA, United States

**Keywords:** adipose tissue, Tregs, immunometabolism, tissue–Treg precursor, obesity****

## Abstract

CD4^+^ Foxp3^+^ regulatory T cells (Tregs) not only enforce peripheral tolerance and restrain self-reactive immune responses, but also maintain organismal homeostasis and safeguard the function of parenchymal tissues. A paradigmatic tissue–Treg population resides in the visceral adipose tissue (VAT) and regulates organismal metabolism by interacting with adipocytes and local immunocytes. Compared with their lymphoid-tissue counterparts, VAT–Tregs have a distinct T cell receptor (TCR) repertoire and transcriptional profile, allowing them to maintain and function in the unique tissue microenvironment. However, when, where, and how VAT–Tregs acquire their distinct features and what signals drive their phenotypic diversification have just started to be unraveled. Here we summarize the recent advances in our understanding on the mechanisms of VAT–Treg derivation and differentiation. We discuss the origin and life history of VAT–Tregs, review the identification and characterization of a VAT–Treg precursor population in the secondary lymphoid organs, and highlight a stepwise reprogramming model of VAT–Treg differentiation that involves multiple stages at distinct locations. Lastly, we discuss whether a similar process may also be involved in the differentiation of Tregs from other non-lymphoid tissues and the imperative questions that remain to be addressed.

## Introduction

Foxp3^+^ regulatory T cells (Tregs) are a subset of CD4^+^ T cells that are crucial for maintaining immune tolerance (1). Humans and mice with a defective Treg compartment due to mutations in the *Foxp3* gene develop a fatal multiorgan autoimmune disorder. For many years, our understanding about Tregs had come mostly from analyses of those residing in the lymphoid organs and how they restrict self-reactive immune responses. Recently, emerging evidences suggest that Tregs also accumulate in a variety of non-lymphoid tissues (NLTs) (e.g., skin, brain, skeletal muscle, colon, and adipose tissue, etc.), display distinct antigen receptor repertoires and transcriptional profiles, and have much broader functions such as promoting tissue homeostasis and regulating organismal metabolism by interreacting with local immune, stromal and parenchymal cells (2, 3).

A paradigmatic “tissue–Treg” population resides in the visceral adipose tissue (VAT) (4). Gain- and loss-of-function studies from multiple groups showed that VAT–Tregs from mice younger than 30–40 weeks of age maintain adipose-tissue homeostasis, restrict inflammation, and promote insulin sensitivity (5–8). In contrast, one study suggested that VAT–Tregs may also drive insulin resistance in mice older than 55 weeks of age (9). A similar population of Tregs has been identified in the omental fat from humans. In several studies, the accumulation of fat Tregs was inversely correlated with the body mass index and fasting glucose levels, while a few other studies found increased Tregs in VAT from obese individuals (10). This discrepancy may arise from the different timing or severity of obesity, as Tregs often accumulate in response to acute inflammation to counteract immune pathology.

Compared with their lymphoid-organ counterparts, VAT–Tregs are unique in several aspects. First, they show a clear sexual dimorphism. Tregs from the epidydimal adipose depot (eVAT) of male C57BL/6 (B6) mice start to accumulate at 10-15 weeks of age and can reach as high as 40–80% of the CD4^+^ T-cell compartment by 20–30 weeks (5). In contrast, they are present at a much lower frequency (10–15% of CD4^+^ T cells) and numbers in the ovarian adipose depot (oVAT) from female mice at similar ages (11, 12). Recent studies showed that compared with females, male mice have elevated basal VAT inflammation and host a unique VAT–Treg supporting stromal-cell population in a sex-hormone-dependent manner, which promote accumulation of eVAT–Tregs as a feedback mechanism to limit the heightened inflammation (12, 13). Second, unlike lymphoid-organ Tregs, eVAT–Tregs display a significantly clonal expanded T cell receptor (TCR) repertoire, indicative of specific antigen recognition in the local tissue (5, 14). Tregs expressing a transgenic (tg) TCR derived from an expanded eVAT–Treg clone, vTreg53, but not polyclonal Tregs, preferentially accumulate in eVAT at steady state and following adoptive transfer, indicating that TCR specificity is a major driver for the accumulation of eVAT–Tregs (11). The TCR clonality of oVAT–Tregs from females and whether TCR specificity is required for their maintenance are less clear. Lastly, eVAT–Tregs are transcriptionally distinct from their lymphoid-organ counterparts, with thousands of genes differentially expressed. In contrast, Tregs from oVAT of female mice are phenotypically much similar to lymphoid-organ Tregs (12). The unique transcriptome of eVAT–Tregs is mainly driven by the nuclear hormone receptor PPARγ, a “master” regulator of adipocyte differentiation. Mice with Treg-specific ablation of PPARγ have a selective loss of the eVAT, but not lymphoid-organ Treg compartment, while transduction of PPARγ together with Foxp3 in splenic CD4^+^ Foxp3^-^ T conventional cells (Tconvs) recapitulate an eVAT–Treg phenotype (15). In addition to PPARγ, accumulation of eVAT–Tregs depends on a variety of factors including Foxp3, BATF, IRF4, BLIMP1, ID2, and the IL-33-ST2 axis (6, 11, 12, 16, 17). For the purpose of this review, we will focus our discussions on the derivation and differentiation of eVAT–Tregs (referred as VAT–Tregs in the following sections).

A fundamental, but difficult to answer, set of questions about VAT–Tregs and NLT Tregs in general relates to their origin, derivation, and differentiation: Where do VAT–Tregs come from? How are they recruited to the adipose tissue and sustained therein? When, where and how do they take on their distinct phenotypes? Here we review recent progress on these important issues. We highlight a stepwise, multi-site model for the derivation and differentiation of VAT–Tregs and discuss whether a similar process may also govern differentiation of Tregs from other NLTs.

## Origin of VAT–Tregs

Foxp3^+^ Tregs can either differentiate in the thymus (tTregs) or be converted from Foxp3^-^ T conventional CD4^+^ T cells (Tconvs) in the periphery (pTregs) (18). Using immunologic and genetic approaches, several studies showed that the VAT–Treg population is derived from the thymus. First, single-cell TCR sequencing analysis in either wild type B6 mice or a mouse line with limited TCR diversity revealed little overlap between VAT–Tregs and Tconvs from VAT or lymphoid organs (5, 14). Second, VAT–Tregs express high levels of Helios and Nrp-1 (14), which are purported markers that distinguish tTregs from pTregs. Third, VAT–Treg do not show preferential expression of previously defined gene signatures of pTregs (14). Lastly, following adaptive transfer, polyclonal Tconvs or vTreg53 TCR-tg Tconvs fail to give rise to VAT–Tregs (11). Interestingly, studies using thymectomy and Treg-punctual ablation approaches indicate that the generation and seeding of Treg in VAT occur primarily in the first weeks of life (reviewed in (4). It remains to be determined what perinatal factors facilitate the selection, differentiation, and/or trafficking of VAT–Tregs.

## Stepwise, Multi-Site Acquisition of the Unique VAT–Treg Phenotype

A key question relates to where and how VAT–Tregs acquire their unique phenotype. Two different scenarios have been proposed (2). Precursors of VAT–Tregs may have already acquired their phenotype or have been pre-committed as they emerge from the thymus, possibly due to unique modes of TCR-antigen recognition in the thymus. Alternatively, VAT–Tregs may only exhibit their distinctive characteristics after installed within the lipid-rich tissues, potentially in response to particular local cues. Addressing these different scenarios, however, had been challenging due to the rarity and inaccessibility of VAT–Tregs, along with the lack of cell transfer system and lineage-tracing tools to study this unique population. Employing a TCR-tg mouse line based on an expanded VAT–Treg clone vTreg53, and a PPARγ-Tdtomato (Tdt) reporter mouse line that fluorescently marks the expression of a key transcription factor for VAT–Tregs, a recent study uncovered a novel stepwise, multi-site scenario for the acquisition of the definitive VAT–Treg phenotype (11).

**Figure 1:**
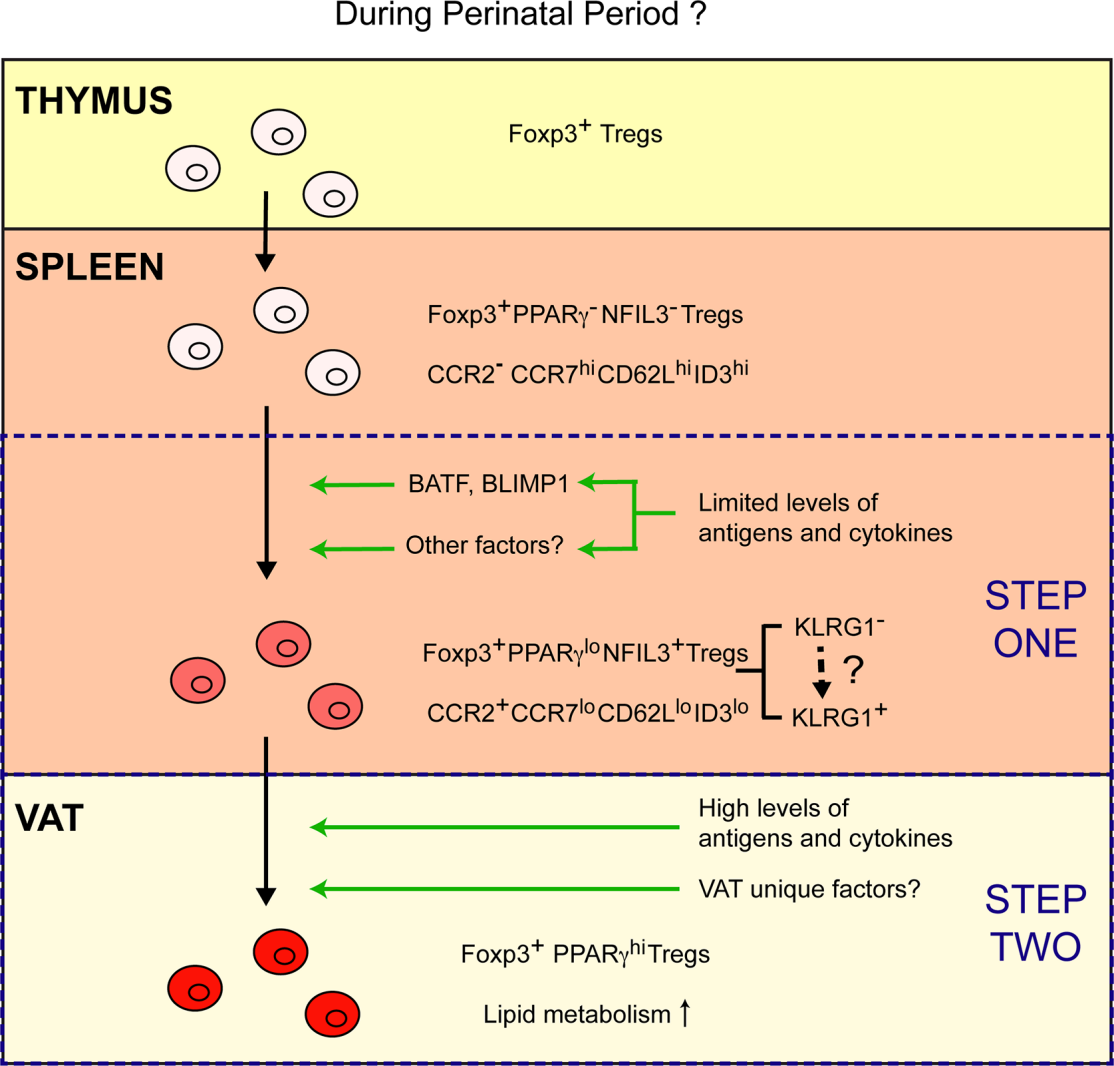
A stepwise, multi-site model of VAT–Treg differentiation. Emerging from the thymus, Tregs lack typical characteristics of VAT–Tregs and are PPARγ^-^ NFIL3^-^. A small fraction of Tregs in the spleen differentiate into PPARγ^lo^ NFIL3^+^ VAT–Treg precursor cells. Limited amount of TCR and cytokine stimulation might drive the expression of BATF, BLIMP1 and other potential factors that are required for the generation of PPARγ^lo^ NFIL3^+^ Tregs in the spleen. Differentiation of the PPARγ^lo^ NFIL3^+^ precursor population is associated with the induction of a Treg activation program, upregulation of CCR2, and downregulation of CCR7, CD62L, and ID3. Such reprogramming enables these cells to exit lymphoid organs and surveil non-lymphoid tissues. Once the precursor cells are installed in the VAT upon recognition of certain local antigens, they can fully mature into PPARγ^hi^ VAT–Tregs that upregulated a lipid metabolism program in response to high levels of TCR and cytokine stimulation, in combination with other unknown VAT unique factors.

In this study, it is found that vTreg53 Tregs emerge from the thymus lacking detectable expression of PPARγ and other typical characteristics of VAT–Tregs, while they uniformly express PPARγ at high levels in the VAT. Unexpectedly, a small fraction (~10%) of Tregs from the secondary lymphoid organs (SLOs), particularly those from the spleen, also express PPARγ, although at a lower level than VAT–Tregs. RNA-seq analysis of this PPARγ^lo^ Treg population further revealed that they have partially acquired the VAT–Treg phenotype, upregulating gene signatures of Treg activation (*e.g.*, *Cd44, Klrg1, Prdm1, Batf*), cytokine sensing (*e.g., Il1rl1, Il9r*), and migration to NLTs (*e.g.*, *Ccr2, Ccr3, Ccr8*), while downregulating genes associated with lymphoid tissue trafficking (*e.g.*, *Ccr7, Sell*) and resting state (*e.g.*, *Tcf7, Lef1*). This reprogramming allows the PPARγ^lo^ Treg to exit lymphoid tissues and surveil NLTs. However, at this stage, these splenic PPARγ^lo^ cells are not yet mature VAT–Tregs since they lack certain hallmarks of mature VAT–Tregs, such as the expression of many transcripts encoding lipid metabolic enzymes (5). Transfer experiments using vTreg53 TCR-tg mice demonstrated that the PPARγ^lo^ population is derived from the PPARγ^-^ Treg compartment in the spleen, and that they efficiently give rise to PPARγ^hi^ population in VAT. Epigenetic profiling of the PPARγ^lo^ splenic Treg population further argues that these cells are precursors of VAT–Tregs, rather than VAT–Tregs recirculating through the lymphoid organs, as the open chromatin landscape of the splenic PPARγ^lo^ population is much more similar to that of PPARγ^-^ splenic Tregs than PPARγ^hi^ VAT–Tregs. Lastly, single-cell RNA-seq analysis could identify corresponding populations of splenic Tregs (~10%) that show weak induction of VAT–Treg signature genes. Therefore, this study strongly argues that the distinctive VAT–Treg phenotype is acquired *via* a stepwise, multi-site process (**Figure 1**).

## Differentiation of Tregs From Other Non-Lymphoid Tissues

Using different markers and single-cell genomics, several recent studies also strongly support this stepwise, multi-site model, and suggest that differentiation of Tregs from other NLTs may also follow a similar trajectory.

As a major barrier site, skin hosts a large population of Tregs that are important for suppressing local inflammation and promoting tissue-specific functions such as hair regeneration and wound healing (19). Similar to VAT–Tregs, most skin Tregs are Helios^+^ Nrp1^+^ and are derived from the thymus (20). In a recent study, Rudensky and colleagues identified a unique Treg subset expressing CD49 that is enriched in peripheral blood and skin-draining lymph nodes and survey the skin (21). These CD49b^+^ Tregs are derived from the CD49b^-^ Treg population in the SLOs, are dependent on TCR signaling for their generation, and express significantly higher levels of migratory and homing receptors to the skin. Using single-cell transcriptomics, Teichmann and colleagues also identified specific subpopulations of Tregs in the skin draining lymph nodes that exhibit certain skin-Treg like features, including upregulation of *Itgb1* and *Cxcr3* (22). Future studies using transfers of antigen specific Tregs or lineage tracing systems are needed to definitely establish whether these unique populations of Tregs in the skin draining lymph nodes are indeed precursors of skin Tregs.

Colon is another location where Tregs are enriched. Unlike VAT and skin, colon Tregs are composed of a RORγt^+^ pTreg population that controls tolerance to the gut microbiota (23, 24) and a GATA3^+^ ST2^+^ tTreg population involved in tissue repair (25). For the differentiation of RORγt^+^ pTregs, several studies showed that Foxp3^-^ Tconv cells convert to Foxp3^+^ Tregs in the mesenteric lymph nodes, upregulate gut-homing receptors such as CCR9 and α4β7, and then traffic to the intestinal lamina propria, where they turn into RORγt^+^ cells in response to the gut microbiota (reviewed in (20)), although a recent study showed that the induction of Foxp3 and RORγt occurs almost simultaneously (26). The differentiation trajectory of the GATA3^+^ ST2^+^ tTreg population in the colon is less understood, but a recent study suggests that these cells may also be derived from specific precursor Treg populations in the mesenteric lymph nodes (27).

Consistent with these results, one study found that downregulation of a transcriptional regulator ID3 in splenic Tregs is associated with the acquisition of a “tissue–Treg” phenotype (28). Of note, using NFIL3 and KLRG1 as markers, a recent study identified a common precursor population for ST2-expressing NLT Tregs and further delineated the these precursors into two stages (27). In this model, NFIL3^-^KLRG1^-^ resting Tregs from the secondary lymphoid organs can engender two NFIL3^+^ Treg populations that are either KLRG1^-^ or KLRG1^+^. While it is proposed by this study that the NFIL3^+^KLRG1^+^ population is more advanced and can be derived from the NFIL3^+^KLRG1^-^ population, further lineage tracing studies are required to determine the exact relationship between these two precursor populations and how they give rise to Tregs from different NLTs.

Do precursor populations identified by different markers in these studies represent same or different populations? Transcriptional analysis from bulk cell populations indicates that at least some of these precursor populations are overlapping. For example, compared with their PPARγ^-^ counterparts, PPARγ^lo^ splenic Tregs express significantly higher levels of *Nfil3*, *Klrg1* and lower levels of *Id3* (11). Similarly, NFIL3^+^ splenic Tregs express higher levels of *Pparg* comparing with NFIL3^-^ cells (27). Further studies using single cell approaches are required to determine the exact relationship among these precursor populations and whether there are different sub-populations of progenitor cells that preferentially give rise to Tregs from different NLTs. It also remains to be determined whether such a stepwise, multisite scenario could also be applied to the differentiation of “tissue Tregs” in humans. Of note, one recent study showed that the core identity of Tregs from NLTs is largely conserved between human and mouse, and sharing of TCR clones between Tregs from the blood and skin of humans has been observed (22). This result indicates that human “tissue Tregs” may also be derived in a stepwise manner. Collectively, results from these studies strongly argue that the unique phenotypes of VAT–Tregs, and likely other NLT Tregs, are first partially acquired in SLOs as one (or two) precursor stage(s), and then fully established in the NLTs (**Figure 1**).

## Discussion

### What Are the Intrinsic Factors That Are Required for the Induction/Maintenance of Precursors for Tregs From VAT and Other NLTs?

Given its importance in programming the unique phenotype of VAT–Tregs (15), PPARγ appears to be one of the top candidates for inducing the VAT–Treg precursors in the spleen. Examining the precursor population by surrogate markers such as NFIL3 and KLRG1 in *Pparg^flox^ Foxp3-Cre* mice that lack PPARγ specifically in Tregs will be informative to determine whether PPARγ is just a marker for VAT–Treg precursors or indeed required for the induction/maintenance of this population. By performing ATAC-seq and motif analysis on the NFIL3^+^KLRG1^-^ and NFIL3^+^KLRG1^+^ precursor cells, Feuerer and colleagues identified BATF as a potential driver for the differentiation of “tissue–Treg” precursors (27). Indeed, in *Batf^-/-^* mice, “tissue–Treg” precursors marked by KLRG1 and PD1 expression fail to develop in the spleen. Using bone marrow chimera and adoptive cell transfer experiments, they further showed that the effect of *Batf* ablation is Treg intrinsic. However, since both KLRG1^-^ and KLRG1^+^ precursor cells are reduced in the absence of BATF, it remains to be determined whether any factors may control each of the KLRG1^-^ and KLRG1^+^ precursor cells specifically. Lastly, a recent study showed that BLIMP1 might also be involved in establishing the VAT–Treg–precursor compartment, since mice lacking BLIMP1 specifically in Tregs also showed a reduction of KLRG1^+^CCR2^+^ Tregs in the spleen (12). In the future, it will be important to address whether and how these different transcription factors interact with each other to establish the “tissue–Treg” precursors.

### What Are the Signals and Cells That Drive the Induction of Precursors for Tregs From VAT and Other NLTs?

Several lines of evidence suggest that TCR signaling is one of the drivers for the induction of VAT–Treg precursors. First, VAT–Treg precursor cells show enrichment of gene signatures associated with Treg activation (11). Second, double-sorted PPARγ^-^ splenic Tregs can give rise to PPARγ^+^ cells by stimulation with anti-CD3/28-coated beads *in vitro* (11), indicating that TCR activation can induce *Pparg* expression. Lastly, when vTreg53 TCR-tg PPARγ^-^ splenic Tregs were transferred into wild type B6 recipients, a fraction of them gave rise to PPARγ^lo^ splenic Tregs, but this did not occur in the presence of an anti-MHCII antibody that blocks TCR activation (11), suggesting that TCR signaling is required for the induction of VAT–Treg precursors. However, it is not clear whether this stimulation is mediated by specific recognition of cognate antigens or generated by tonic signals while Tregs circulate through secondary lymphoid organs. Using yeast-display screening, several surrogate peptides that can stimulate the vTreg53 TCR with different signaling potency have been recently identified (29). It would be interesting to determine whether stimulation with these peptides can promote the induction of VAT–Treg precursors in the spleen, and whether TCR signaling strength affects the efficiency of such induction.

In addition to TCR, several cytokines, including IL-33, IL-4, and IL-6, have been suggested to play a role in the induction/maintenance of precursors for VAT–Tregs and other NLT Tregs. Addition of IL-33, IL-4, and IL-6, either alone or in combination, in the presence of TCR stimulation, could enhance the generation of PPARγ^+^, NFIL3^+^, or BLIMP1^+^ Tregs and promote the induction of a “tissue–Treg” program *in vitro* (11, 12, 27). However, whether these cytokines function by promoting the induction of new “tissue–Treg” precursors, or by expanding existing “tissue–Treg” precursors are unknown. It is also worth mentioning that without loss-of-function experiments, one cannot conclude whether these cytokines are indeed required for the generation of “tissue–Treg” precursors *in vivo*. Analysis of mice lacking the receptors of these cytokines specifically in Tregs should help address this issue. Lastly, as certain stromal and immune populations are specialized in presenting antigens and producing some of these cytokines in defined locations, further investigation is needed to characterize the interactions between these different cell types with Tregs in the secondary lymphoid organs, and how these interactions control the induction/maintenance of “tissue–Treg” precursors. The competition for such interactions could also potentially explain why the precursor population is usually held below 10% of the splenic-Treg compartment.

### What Is the Evolutionary Advantage of Having a “Tissue–Treg” Precursor Population in the SLOs?

It is currently unclear why there needs to be a precursor stage for “tissue–Tregs” in the SLOs. One property of the “tissue–Treg” precursors is that they “turn on” multiple migration molecules (*e.g.*, *Ccr2, Ccr3, Ccr8*) that guide them into non-lymphoid tissues and “turn off” certain molecules (*e.g.*, *Ccr7, Sell*) that trap them in the SLOs. Therefore, it is possible that only Tregs that have received certain instructive signals (*e.g.*, TCR, cytokines, etc.) can exit lymphoid organs and surveil non-lymphoid tissues. Comparing with an alternative scenario in which all Tregs circulating through non-lymphoid tissues looking for antigens, generation of a precursor population in the lymphoid organs could be much more cost-effective so that only cells that have certain unique properties (*e.g.*, specificity to tissue antigens, ability to sense particular environmental cues) can become “tissue–Tregs”. In addition, since “tissue–Tregs” are highly reprogramed, with hundreds to thousands of genes induced or suppressed, acquisition of a partial phenotype/transcriptome in the SLOs could better prepare the cells so that they can quickly adapt to the new environment and function effectively once they are installed in the NLTs.

In summary, identification of a stepwise, multi-site scenario for the differentiation of Tregs from VAT and other NLTs opens up many exciting new directions for research in this area. Addressing these questions will not only improve our understanding on the basic biology of VAT–Tregs and “tissue–Tregs” in general, but also aid us to design novel strategies to modulate “tissue–Tregs” specifically to treat various inflammatory or metabolic diseases.

## Author Contributions

CL conceptualized the review. CL and PS prepared the manuscript. All authors contributed to the article and approved the submitted version.

## Funding

This work was supported by the startup fund from Emory University School of Medicine for CL.

## Conflict of Interest

The authors declare that the research was conducted in the absence of any commercial or financial relationships that could be construed as a potential conflict of interest.
